# Premature atrial and ventricular contractions detected on wearable-format electrocardiograms and prediction of cardiovascular events

**DOI:** 10.1093/ehjdh/ztad007

**Published:** 2023-02-03

**Authors:** Michele Orini, Stefan van Duijvenboden, William J Young, Julia Ramírez, Aled R Jones, Andrew Tinker, Patricia B Munroe, Pier D Lambiase

**Affiliations:** Institute of Cardiovascular Science, University College London, Gower Street, London WC1E 6BT, UK; MRC Unit for Lifelong Health and Ageing, Institute of Cardiovascular Science, University College London, London WC1E 6BT, UK; Barts Heart Centre, St Bartholomew’s Hospital, West Smithfield, London EC1A 7BE, UK; Institute of Cardiovascular Science, University College London, Gower Street, London WC1E 6BT, UK; Clinical Pharmacology and Precision Medicine, Faculty of Medicine and Dentistry, William Harvey Research Institute, Queen Mary University of London, Charterhouse Square, London EC1M 6BQ, UK; Nuffield Department of Population Health, University of Oxford, Oxford OX3 7LF, UK; Barts Heart Centre, St Bartholomew’s Hospital, West Smithfield, London EC1A 7BE, UK; Clinical Pharmacology and Precision Medicine, Faculty of Medicine and Dentistry, William Harvey Research Institute, Queen Mary University of London, Charterhouse Square, London EC1M 6BQ, UK; Clinical Pharmacology and Precision Medicine, Faculty of Medicine and Dentistry, William Harvey Research Institute, Queen Mary University of London, Charterhouse Square, London EC1M 6BQ, UK; Aragon Institute of Engineering Research, University of Zaragoza and Centro de Investigación Biomédica en Red, Bioingeniería, Biomateriales y Nanotecnología Zaragoza, C/ de Mariano Esquillor Gómez, Zaragoza 50018, Spain; Barts Heart Centre, St Bartholomew’s Hospital, West Smithfield, London EC1A 7BE, UK; Clinical Pharmacology and Precision Medicine, Faculty of Medicine and Dentistry, William Harvey Research Institute, Queen Mary University of London, Charterhouse Square, London EC1M 6BQ, UK; Clinical Pharmacology and Precision Medicine, Faculty of Medicine and Dentistry, William Harvey Research Institute, Queen Mary University of London, Charterhouse Square, London EC1M 6BQ, UK; Clinical Pharmacology and Precision Medicine, Faculty of Medicine and Dentistry, William Harvey Research Institute, Queen Mary University of London, Charterhouse Square, London EC1M 6BQ, UK; Institute of Cardiovascular Science, University College London, Gower Street, London WC1E 6BT, UK; Barts Heart Centre, St Bartholomew’s Hospital, West Smithfield, London EC1A 7BE, UK

**Keywords:** Wearable ECG, Premature contractions, Arrhythmias, Heart failure, Atrial fibrillation

## Abstract

**Aims:**

Wearable devices are transforming the electrocardiogram (ECG) into a ubiquitous medical test. This study assesses the association between premature ventricular and atrial contractions (PVCs and PACs) detected on wearable-format ECGs (15 s single lead) and cardiovascular outcomes in individuals without cardiovascular disease (CVD).

**Methods and results:**

Premature atrial contractions and PVCs were identified in 15 s single-lead ECGs from *N* = 54 016 UK Biobank participants (median age, interquartile range, age 58, 50–63 years, 54% female). Cox regression models adjusted for traditional risk factors were used to determine associations with atrial fibrillation (AF), heart failure (HF), myocardial infarction (MI), stroke, life-threatening ventricular arrhythmias (LTVAs), and mortality over a period of 11.5 (11.4–11.7) years. The strongest associations were found between PVCs (prevalence 2.2%) and HF (hazard ratio, HR, 95% confidence interval = 2.09, 1.58–2.78) and between PACs (prevalence 1.9%) and AF (HR = 2.52, 2.11–3.01), with shorter prematurity further increasing risk. Premature ventricular contractions and PACs were also associated with LTVA (*P* < 0.05). Associations with MI, stroke, and mortality were significant only in unadjusted models. In a separate UK Biobank sub-study sample [UKB-2, *N* = 29,324, age 64, 58–60 years, 54% female, follow-up 3.5 (2.6–4.8) years] used for independent validation, after adjusting for risk factors, PACs were associated with AF (HR = 1.80, 1.12–2.89) and PVCs with HF (HR = 2.32, 1.28–4.22).

**Conclusion:**

In middle-aged individuals without CVD, premature contractions identified in 15 s single-lead ECGs are strongly associated with an increased risk of AF and HF. These data warrant further investigation to assess the role of wearable ECGs for early cardiovascular risk stratification.

## Graphical Abstract

**Graphical Abstract:**
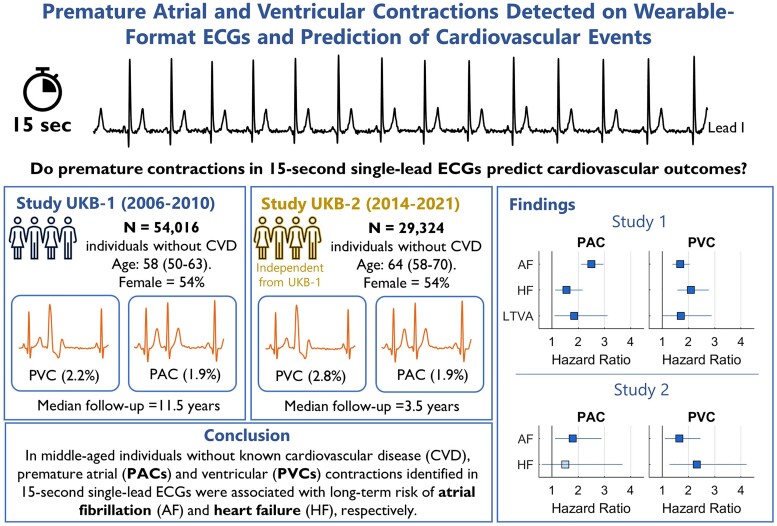

## Introduction

Premature contractions, also known as ectopic beats, occur independently of the heart’s physiological pacemaker and are common in healthy individuals. Depending on their origin, they are classified as premature atrial or ventricular contractions (PACs and PVCs, respectively). Frequent premature contractions (e.g. >1000/day) are associated with an increased risk of adverse cardiovascular events including atrial fibrillation (AF), heart failure (HF), stroke, and mortality in individuals with and without cardiovascular disease (CVD).^1–4^ Monitoring premature contractions requires an electrocardiogram (ECG) recorded either using long-term ambulatory devices (from 24 h to 14 days) or during an exercise stress test. Although the ECG is one of the most widely prescribed and affordable medical tests, its current use is restricted to the clinical setting. The standard 12-lead ECG requires the application of electrodes by a trained health practitioner, while long-term (24 h to 14 days) ambulatory monitoring^5^ requires expensive devices and expert revision on dedicated analytical platforms. Wearable and mobile devices allowing ECG self-recording are becoming increasingly available and affordable. These include smart watches (e.g. Apple Watch 4)^6–8^ and ECG monitors compatible with mobile phones (e.g. Alivecor Kardia),^7,9^ which have shown potential for detecting AF^6,9^ and measuring cardiac intervals.^8^ Most of these devices use single-lead recordings of short duration (typically 10–30 s for Lead I).

A limited number of population-based studies have suggested that the presence of a premature contraction on a standard 10–15-s 12-lead ECG is associated with adverse outcomes.^10–12^ The data are, however, limited, and a recent systematic review has concluded that the evidence for establishing a link between PAC on a standard 12-lead ECG and AF or mortality is insufficient.^1^

The aim of this study was to investigate the association of PACs and PVCs identified in ECGs recorded in 15 s single-lead ECGs (a similar configuration used by popular wearable devices) with future cardiovascular events. We hypothesized that in participants without CVD, the presence of at least one PAC or PVC in a 15 s resting ECG would indicate a high ectopic burden and would be associated with an increased risk of AF and HF, respectively.

We used a large UK Biobank sub-study (UKB-1, recruited between 2006 and 2013) including 54 016 participants without known CVD with over 11 years of follow-up and comprehensive outcome measures. We used a second UK Biobank sub-study (UKB-2, initiated in 2014) including 29 324 participants not studied in UKB-1 for independent validation.

## Methods

### Study design

UK Biobank participants who underwent an ECG recording during the initial assessment visit in 2006–10 and the first repeat assessment visit in 2012–13 were included in the primary analysis (cohort UKB-1). Participants who underwent a standard 12-lead ECG recording during the imaging visit (from 2014) or during the first repeated imaging visit (from 2019) were included in the validation cohort (UKB-2). The UK Biobank study has approval from the North West Multi-Centre Research Ethics Committee, and all participants provided informed consent.^13^ In the UKB-1 study, Lead I ECG was measured for 15 s in a sitting position (protocol available online)^14^ while in UKB-2, a standard 15 s 12-lead ECG was measured in supine position (protocol available online)^15^ and only Lead I was analysed in this study for consistency with UKB-1. All recordings were taken with a GE CardioSoft system with a sampling frequency equal to 500 Hz. If a participant was recruited in both UKB-1 and UKB-2, only data from UKB-1 were included in this study to ensure that cohorts did not overlap, and analyses were performed on independent data sets.

**Figure 1:**
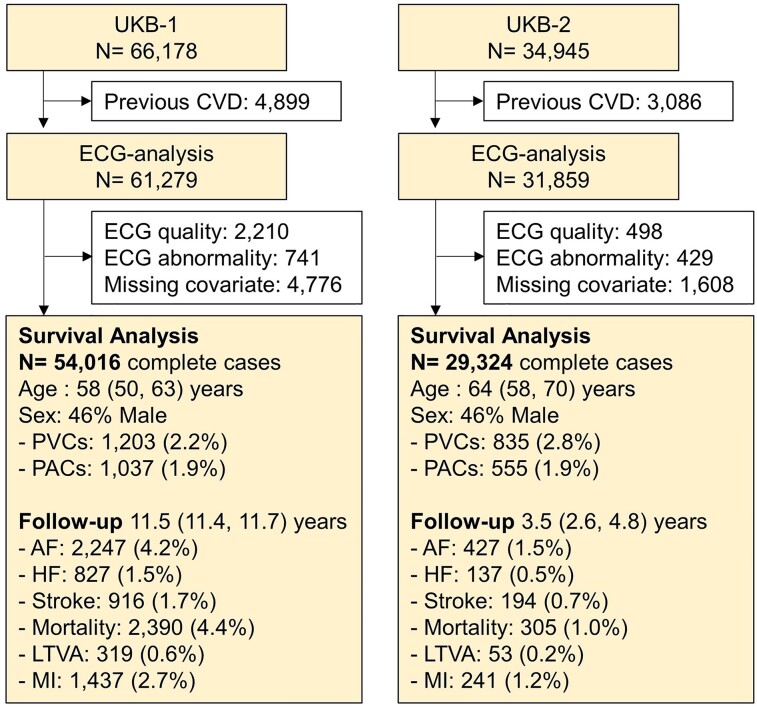
Flow diagram of the study. AF, atrial fibrillation; CVD, cardiovascular disease; HF, heart failure; LTVA, life-threatening ventricular arrhythmia; MI, myocardial infarction; PVCs and PACs, premature ventricular and atrial contractions. Electrocardiogram abnormality includes bundle branch block morphology, sinus node dysfunction, and atrial fibrillation.

*Figure 1* provides an overview of the study. Electrocardiogram recordings were available for analysis in 66 178 and 34 945 individuals in UKB-1 and UKB-2, respectively. Electrocardiograms from individuals with CVD (*N* = 4899 and *N* = 3086 for UKB-1 and UKB-2, respectively) were excluded and not analysed. A participant was considered as having CVD if evidence of CVD was identified from hospital episode statistics or if CVD was self-reported (including inability to exercise in UKB-1 due to an established cardiovascular diagnosis). Previous diagnoses of cardiovascular events were identified through Hospital Episode Statistics and included ischaemic, congenital, and valvular heart disease, cardiomyopathies, HF, atrial and ventricular arrhythmias, stroke, vascular disease, and myocarditis (see Supplementary material online, *Table S1* for the full list of codes). Electrocardiograms were then processed as described in the following section, and recordings were excluded from statistical analysis if (i) the ECG signal quality was insufficient to discriminate between sinus and abnormal rhythm upon visual inspection; (ii) the ECG recording was considered abnormal, which included bundle branch block morphology, sinus node dysfunction, and AF; and (iii) the information about risk factors included in the statistical modelling was missing.

### Electrocardiogram analysis

Identification of PACs and PVCs was expert-based, but aided by automated signal processing tools, as in previous studies,^16–18^ and machine learning algorithms implemented in MATLAB. The expert-based classification was performed upon visual inspection and mainly informed by the morphology of the heartbeats (PVCs show distinctive QRST morphologies, with usually wider QRS complexes and large T waves) and by prematurity (PACs show similar morphology as sinus beats but occur much earlier than expected and are often but not always followed by a compensatory pause). Since premature contractions occur, by definition, earlier than normal beats, recordings showing heart rate irregularity indicating abrupt and large changes in the inter-beat interval were selected as potentially showing PVCs or PACs. Heart rate irregularity was measured using the standard deviation of the successive differences in the RR interval. A machine learning algorithm previously developed by our group was subsequently used on the entire data set to specifically identify PVCs (see Supplementary material online, *Methods*). The flowchart indicating the procedure for ECG classification is reported in Supplementary material online, *Figure S1*. Electrocardiograms were split into two groups: Group A included participants showing top 30% heart rate irregularity or PVCs (*N* = 19 561) and Group B included the rest (lower 70% of heart rate irregularity and no PVCs). All ECGs in Group A were reviewed on a graphical user interface (see Supplementary material online, *Figure S2*) and classified into: Normal sinus rhythm, PACs, PVCs, abnormal rhythm (AF or sinus node dysfunction), or undetermined due to low signal quality (noisy ECGs). The initial visual revision was performed by one expert operator, with diagnoses of PVC and PAC confirmed by a second expert. Ambiguous cases (*N* = 292) were discussed with a 3rd expert to reach a consensus. Only recordings showing sinus rhythm, PACs or PVCs were included in the analysis, while abnormal or noisy ECGs were excluded. Since all recordings labelled as containing PACs and PVCs were visually reviewed, and since expert-based detection of PACs and PVCs is very accurate in most cases, this approach minimizes false-positive detections. To assess the false-negative rate, 15 000 ECGs were randomly selected from Group B and manually reviewed. Of these, five showed a PVC and one showed a PAC, which corresponds to a false-negative rate < 0.04%, and one ECG was re-labelled as abnormal (AF) and two were re-labelled as noisy. Because of the low false-negative rate, the remaining ECGs from Group B (*N* = 26 142) were considered normal without visual inspection.

A similar approach was used to analyse data from UKB-2 (see Supplementary material online, *Figure S1*).

### Statistical analysis

Data distribution is reported as median, interquartile range, and results of survival analysis as hazard ratio (HR) with 95% confidence interval (CI). Information about death was obtained through NHS Digital (England and Wales) and from the NHS Central Register (Scotland). Outcomes were derived from Hospital Episode Statistics codes as in previous studies^17,18^ (see Supplementary material online, *Table S2*). Primary outcomes were AF and HF. Secondary outcomes included MI, stroke and transient ischaemic attacks, life-threatening ventricular arrhythmia (LTVA), and mortality. Survival analysis was performed using Cox regression models in MATLAB R2019b. Premature atrial contraction and PVC were included in the same model and adjusted for age, sex, body mass index, hypertension, smoking, LDL cholesterol, diabetes, and use of beta-blockers. In UKB-2, data on cholesterol were not available and the model was adjusted for age, sex, body mass index, hypertension, smoking, diabetes, and use of beta-blockers. Differences between HRs were assessed using the *survcomp* package in R 3.6.

In sensitivity analyses, associations with outcomes were assessed after (i) excluding participants using beta-blockers and (ii) excluding participants with any cardiovascular diagnosis (see Supplementary material online, *Table S1*) within 1 year from baseline to attempt to reduce reverse causality. Furthermore, to assess potential selection bias, exclusions because of missing covariates and low ECG signal quality were used as exposures and their association with outcomes was assessed. Finally, competing risk analysis, used for confirming associations with LTVA, was implemented with R package *cmprsk*.

## Results

In UKB-1, statistical analysis was conducted on 54 016 complete cases. The median (interquartile age) age was 58 (50–63) years and 54% were women. At least one PAC or PVC was observed in 1.9 and 2.2% of participants, respectively. Compared with participants without premature contractions at baseline, those with at least one premature contraction were older, had a higher body mass index, and were more frequently male, and showed a higher prevalence of Type 2 diabetes and hypertension (*Table 1*). No difference was found in smoking status, use of beta-blockers and LDL cholesterol. After a median follow-up of 11.5 years (interquartile range 11.4–11.7 years), the proportion of participants with outcomes was: 4.4% (mortality), 4.2% (AF), 2.7% (MI), 1.7% (stroke), 1.5% (HF), and 0.6% (LTVA). All outcomes were significantly more frequent in participants with PACs or PVCs vs. those with no premature contractions (*Table 1*).

**Table 1:** Baseline characteristics of the participants by the presence of premature atrial and ventricular contractions and incidence of outcomes

	No PAC	PAC	*P*-value	No PVC	PVC	*P*-value
	52 979 (98.1%)	1037 (1.9%)		52 813 (97.8%)	1203 (2.2%)	
Age (years)	58.0 (50.0–63.0)	63.0 (58.0–66.0)	**8.6E−72**	58.0 (50.0–63.0)	62.0 (56.0–66.0)	**1.8E−53**
Sex (male)	24 308 (45.9%)	574 (55.4%)	**1.7E−09**	24 234 (45.9%)	648 (53.9%)	**4.3E−08**
BMI (kg/m^2^)	26.5 (24.0–29.4)	26.9 (24.5–30.1)	**4.0E−05**	26.5 (24.0–29.4)	27.0 (24.4–30.0)	**4.0E−04**
Hypertension (yes)	27 056 (51.1%)	678 (65.4%)	**4.0E−20**	26 974 (51.1%)	760 (63.2%)	**6.9E−17**
Diabetes (yes)	2313 (4.37%)	70 (6.75%)	**5.5E−04**	2309 (4.37%)	74 (6.15%)	**4.4E−03**
Beta-blockers (yes)	1987 (3.75%)	45 (4.34%)	3.2E−01	1983 (3.75%)	49 (4.07%)	5.4E−01
Smoking (ever)	4470 (8.44%)	93 (8.97%)	5.4E−01	4479 (8.48%)	84 (6.98%)	6.6E−02
LDL (mmol/L)	3.5 (3.0–4.1)	3.5 (2.9–4.1)	6.8E−02	3.5 (3.0–4.1)	3.5 (3.0–4.1)	6.2E−01
AF (yes)	2101 (3.97%)	146 (14.1%)	**3.6E−38**	2138 (4.05%)	109 (9.06%)	**4.7E−14**
HF (yes)	790 (1.49%)	37 (3.57%)	**2.7E−06**	775 (1.47%)	52 (4.32%)	**3.6E−11**
VA (yes)	304 (0.574%)	15 (1.45%)	**1.5E−03**	304 (0.576%)	15 (1.25%)	**6.5E−03**
ACM (yes)	2308 (4.36%)	82 (7.91%)	**2.0E−02**	881 (1.67%)	35 (2.91%)	**2.1E−03**
Stroke (yes)	888 (1.68%)	28 (2.7%)	**3.2E−03**	1392 (2.64%)	45 (3.74%)	**2.3E−02**
MI (yes)	1393 (2.63%)	44 (4.24%)	**1.5E−03**	304 (0.576%)	15 (1.25%)	**6.5E−03**

ACM, all-cause mortality; AF, atrial fibrillation; BMI, body mass index; HF, heart failure; LDL, low-density lipoprotein cholesterol; LTVA, life-threatening ventricular arrhythmia; MI, myocardial infarction; PAC, premature ventricular contraction; PVC, premature atrial contraction.

Statistically significant differences (*P* < 0.05) are highlighted in bold.

### Associations with atrial fibrillation and heart failure

**Figure 2:**
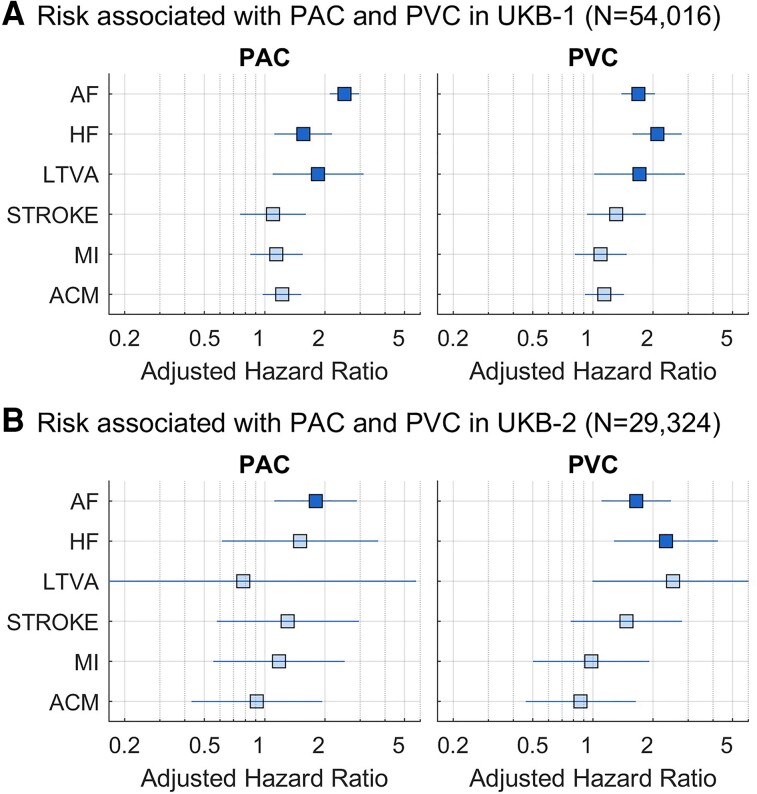
Association of premature atrial contractions and premature ventricular contractions with outcomes in UK Biobank-1 (A) and UK Biobank-2 (B) cohorts. Models are adjusted for risk factors. Markers and bars represent hazard ratio and 95% confidence intervals. ACM, all-cause mortality; AF, atrial fibrillation; HF, heart failure; LTVAs, life-threatening ventricular arrhythmias; MI, myocardial Infarction. Transparent markers show non-significant associations (*P* > 0.05).

In unadjusted models (see Supplementary material online, *Table S3*), PACs and PVCs were significantly associated with both AF (HR, 95% CI: 3.85, 3.26–4.45, *P* < 0.001 and 2.34, 1.93–2.84, *P* < 0.001, respectively) and HF (HR = 2.49, 1.79–3.46, *P* < 0.001 and 3.07, 2.32–4.07, *P* < 0.001, respectively). These associations remained significant after adjustment (*Figure 2*, Supplementary material online, *Table S3*), with PAC showing higher HR for AF (HR = 2.50, 2.11–2.96, *P* < 0.001) and PVC showing higher hazard ratio for HF (HR = 2.09, 1.58–2.78, *P* < 0.001).

**Figure 3:**
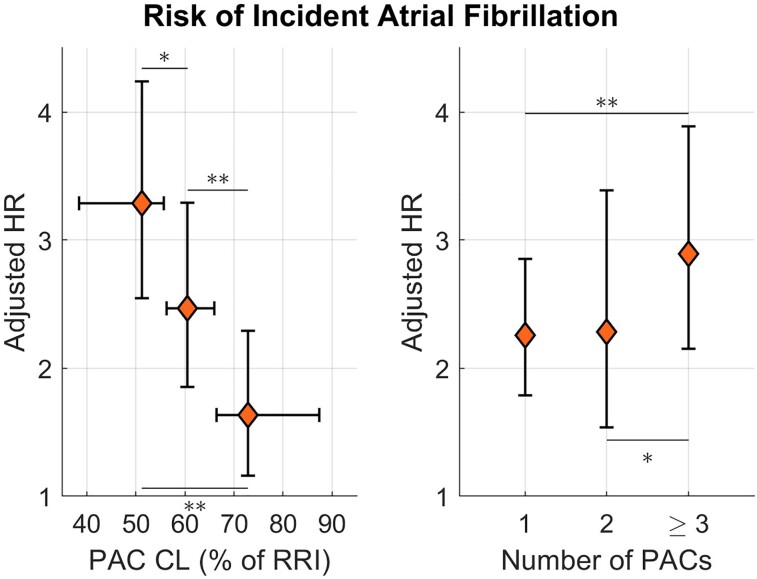
Risk of incident atrial fibrillation (vertical axes) as a function of premature atrial contraction prematurity (left) and premature atrial contraction number (right) in UK Biobank-1. Prematurity is measured as cycle length divided by the median RR interval (RRI) of the five previous beats. HR, hazard ratio; PAC, premature atrial contraction. *P < 0.05; **P < 0.005.

Premature atrial contraction prematurity, measured as the PAC cycle length divided by the median RR interval of the previous five beats, and the number of PACs in the 15 s recordings, showed a dose–response association with incident AF (*Figure 3*). In adjusted Cox models, the HR for AF increased from 1.6 (1.2–2.3) for PAC with longest (3rd tertile) prematurity to 3.3 (2.5–4.2) for PAC with shortest (1st tertile) prematurity, with all pairwise comparisons across tertiles reaching statistical significance. Similarly, participants with three or more PACs showed a higher risk of AF (HR = 2.9, 2.1–3.9) than those with one PAC (2.2, 1.8–2.8). A larger number of PVCs or a shorter PVC prematurity were not increasingly associated with AF or HF risk.

### Associations with secondary outcomes

Premature atrial contractions and PVCs were both associated with LTVA, stroke, MI, and mortality in unadjusted models, with HR ranging between 1.46 and 2.59 (see Supplementary material online, *Table S4*). However, only associations with LTVA remained statistically significant after adjustment (HR = 1.71, 1.01–2.88, *P* = 0.044 for PVCs and HR = 1.85, 1.10–3.12, *P* = 0.020 for PACs, *Figure 2*). The association between PACs and PVCs with LTVA remained significant after using MI and HF as competing risks.

### Associations in men and women

Previous studies have found that the association between premature contractions and cardiovascular outcomes differed in men and women.^19,20^ In this study, all outcomes were more frequent in men than women, with odd ratios exceeding two for most of them. Separate analyses stratifying by sex confirmed that after adjustment, the association between PVCs and HF and between PACs and AF was strongly significant in both men and women. However, some sex-specific differences were found. For instance, the association of PACs with HF or LTVA was only significant in men, whereas the association between PVCs and LTVA was only significant in women (see Supplementary material online, *Table S5*). A significant association between PVCs and mortality was also found in men (HR = 1.33, 95% CI = 1.03–1.71, *P* = 0.030).

### Sensitivity analyses

The exclusion of participants with a cardiovascular event registered within 1 year from ECG recording (*N* = 311) did not attenuate associations between premature contractions and outcomes (see Supplementary material online, *Table S6*). The exclusion of participants taking beta-blockers at baseline had little effect on the association between PACs and AF and between PVC and HF, but it slightly attenuated the association between PACs and HF (HR = 1.40, 0.97–2.02, *P* = 0.07) and between PVC and LTVA (HR = 1.71, 0.99–2.93, *P* = 0.052), which both became borderline non-significant (see Supplementary material online, *Table S7*).

The vast majority of participants excluded because of missing covariates (94% of *N* = 4312) were excluded because LDL cholesterol was not measured. Participants excluded because of missing values were not at higher risk of developing any of the health outcomes.

Participants excluded because of low signal quality (*N* = 2210) showed an increased risk of AF (HR = 1.36, 1.13–1.62, *P* < 0.001), but no increased risk of any other outcome.

### Independent validation in UK Biobank-2

Despite a smaller sample size (*N* = 29 324) and a shorter follow-up (3.5, 2.6–4.8, years), results from UKB-2 were generally in agreement with results from UKB-1. UK Biobank-2 participants were slightly older than UKB-1 participants (64, 58–70 years old). Premature atrial contractions and PVCs were observed in 1.9 and 2.8% of study participants’ 15 s single-lead ECGs, and they were associated with age, being male and having hypertension (see Supplementary material online, *Table S8*). In adjusted models, PACs were associated with AF (HR = 1.80, 95% CI = 1.12–2.89, *P* = 0.016), with shorter prematurity increasing AF risk, and PVCs were associated with HF (HR = 2.32, 95% CI = 1.28–4.22, *P* = 0.006; *Figure 2*, Supplementary material online, *Table S9*). The exclusion of participants taking beta-blockers at baseline did not attenuate these associations. The association between PVCs and LTVA was borderline non-significant after adjusting for risk factors (HR = 2.52, 0.99–6.39, *P* = 0.052).

## Discussion

This study is based on two large independent cohorts from the UKB including middle-aged individuals without CVD (*N* = 54 016 and *N* = 29 324) at baseline with deep phenotyping and comprehensive outcomes. The main result is that the presence of premature contractions in a 15 s single-lead ECG (a format used by popular ECG wearable devices), which was observed in around 2% of the population, was associated with an increased long-term risk of cardiovascular events. The strongest associations, which remained significant after adjusting for standard risk factors in both men and women and were confirmed after sensitivity analyses and independent validation, were found between PVCs and HF (HR = 2.09, 95% CI = 1.58–2.78) and between PACs and AF (HR = 2.52, 95% CI = 2.11–3.01), with PAC prematurity (i.e. shorter coupling interval) and frequency (≥3 PACs in 15 s) further increasing AF risk. This agrees with our hypothesis that individuals with one or more premature contractions in 15 s have a high ectopic burden (an individual with 1 ectopic beat every 15 s would have 5760 ectopic beats per day) and are therefore at higher risk of developing AF and HF. In fact, it is much more likely to observe a premature contraction in a 15 s ECG in individuals with high rather than low ectopic burden. Assuming a constant ectopic rate, the probability of observing an ectopic beat in a 15 s recording increases with the ectopic burden: 0.4% for an individual with 1 ectopic every hour (24 ectopic/day), 2.5% for an individual with 1 ectopic every 10 min (144 ectopic/day), and 25% for an individual with 1 ectopic every minute (1440 ectopic/day). Several possible pathways may explain the association between PACs and PVCs with AF and HF. These include premature contractions serving as triggers for AF and frequent dyssynchronous contractions increasing mechanical stress on atrial and ventricular tissue establishing a substrate for both AF and HF over the long term. Also, as proposed elsewhere,^21^ PACs may be considered as an earlier marker of an atrial cardiomyopathic process leading to the establishment of AF.

Given the predicted increase in the prevalence of AF^22^ and HF,^23^ and their substantial impact on healthcare systems, opportunities for early diagnosis and better preventative strategies are urgently needed. Our findings may have implications for novel mobile health preventive strategies as they suggest that wearable ECG devices enabling self-screening for PACs and PVCs may identify a relatively small number of individuals at higher risk. Most of the wearable ECG Apps focus on AF screening and do not currently detect premature contractions, but our results should encourage investment in this direction. Some wearable ECGs have been shown to record waveforms with morphologic equivalence to the ECG configuration considered in this study (Lead I ECG),^7^ but clinical translation would require prior validation of specific ECG wearable technology against medical graded devices. Further studies are needed to assess how the benefits of mobile healthcare technologies can be balanced against a possible increase in overdiagnosis and overuse of medical services as well as widening digital inequalities with an impact on health outcomes.

In UKB-1, PVCs and PACs were associated with LTVA, and the association remained significant when considering MI and HF as competing risks. This has not been reported before and requires further investigation. Stratification by sex showed that the association between PACs and LTVA was only significant in men, possibly on account of higher incidence in men. The borderline non-significant association between PVCs and LTVA in UKB-2 (*P* = 0.052) may be due to the small number of events (*n* = 53) over a short follow-up period. While an association between PVCs and LTVA may be expected as PVCs are required to trigger LTVAs, the link between PACs and LTVA is less clear. Possible mechanisms include PACs either acting as triggers for ventricular arrhythmia^24^ or reflecting atrial myopathy, potentially affecting the ventricles as well. Alternatively, PAC presence may be considered as a non-specific cardiac risk marker, which would be consistent with previous studies showing that PACs were associated with cardiovascular mortality in individuals without known CVD.^25^

In UKB-1, the association with mortality was only significant in men, while the associations with stroke and myocardial infarction did not remain significant after adjusting for confounding variables.

Community-based studies using 10–15 s ECGs are scant, have often focused on single or few outcomes, and have reported conflicting results.^1,4^ An association between PACs and AF was previously reported in smaller population-based studies using 10–15 s 12-lead ECG (including REGARDS,^26^ ARIC,^11^ CHS,^11^ and IPHS^20^ cohorts), with some of these studies also finding an association with cardiovascular mortality^20,25^ and stroke.^19^ However, a recent systematic review and meta-analysis has concluded that standard 12-lead ECGs provided insufficient evidence of PAC association with future AF or mortality.^1^ Our data complement these studies and provide strong evidence of a link between PACs in 15 s ECGs and incident AF. A recent systematic review on PVCs found four community-based studies reporting on standard 12-lead ECG, with some conflicting results,^3,10,25,27^ and Nguyen *et al.*^11^ have recently shown that PVCs were associated with an increased risk of AF, HF, and mortality in CHS and ARIC cohorts.

While some of our findings, e.g. a significant association between PACs and AF, and between PVCs and HF, as well as the lack of independent association with myocardial infarction, are consistent with previous data,^11,25^ differences may be due to historical trends in CVD (most of the previous studies started in the 1980s and 1990s), geographical and ethnical differences (none of these previous studies were based on a European population), significantly smaller sample size, and inclusion of participants with underlying CVD or rhythm disorders.^11,19,26^ Differences with previous studies based on screening ECGs from the REGARDS cohort, where a modest association with stroke was found for both PACs^19^ and PVCs,^27^ may be due to a much higher prevalence of PACs and PVCs (7.3 and 6.1%, respectively) indicating higher baseline risk in REGARDS when compared with UK Biobank.

This study has several strengths. Firstly, the combined sample size of UKB-1 and UKB-2 was 83 340 individuals. Secondly, the identification of premature contractions was based on expert evaluation supported by advanced computational ECG processing to identify recordings more likely to contain ectopic beats. Our approach was designed to have a zero false-positive rate, as all PACs and PVCs were confirmed by visual inspection, and we estimated a false-negative rate for PACs and PVCs <0.05% by manually reviewing 15 000 ECGs considered normal. The total number of manually reviewed ECGs in UKB-1 was 35 137 ECGs (57% of the total). Thirdly, the UK Biobank is one of the largest contemporary population-based studies, with deep phenotyping and linkage to hospital episode statistics, which allows exclusion of individuals with previous CVD, comprehensive adjustment for risk factors, and follow-up of multiple outcomes. This study also has limitations. As in most studies, ECGs and confounding variables were assessed only at baseline. Hospital episode statistics may underestimate the true incidence of outcomes because of their possible subclinical or unreported manifestations. Self-reported CVD may not accurately reflect the true prevalence of CVD even when combined with diagnoses from hospital episode statistics. There is evidence of ‘healthy volunteer’ selection bias in the UK Biobank,^28^ which may not be representative of the general population. Our finding that individuals for which PAC and PVC detection was inconclusive due to low ECG signal quality were at a higher risk of AF highlights the importance of high-quality recordings.

## Conclusions

In middle-aged individuals without known CVD from two large UK Biobank sub-studies, PACs and PVCs captured from 15 s single-lead ECGs were associated with a long-term risk of AF and HF, respectively, with prematurity and frequency of PACs further increasing the risk of AF. Our results support future studies to investigate the clinical implication of premature heartbeat detection in mobile health population–based risk assessment through ECG wearable devices.

## Supplementary Material

ztad007_Supplementary_Data

## Data Availability

Anonymized data and materials generated in this work will be returned to the UK Biobank and can be accessed upon request.
