# Supplementary material for: Premature atrial and ventricular contractions detected on wearable-format electrocardiograms and prediction of cardiovascular events
Source: Eur Heart J Digit Health. 2023 Feb 3;4(2):112–8. doi: 10.1093/ehjdh/ztad007 (PMC10039429; doi:10.1093/ehjdh/ztad007)
Supplement: ztad007_Supplementary_Data [file ztad007_supplementary_data.zip › Supplementary Material_R1.pdf]

Contents

Supplementary Figure S1 1

Supplementary Figure S2 2

Supplementary Methods 3

Automatic detection of premature ventricular contractions 3

Supplementary Figure S1

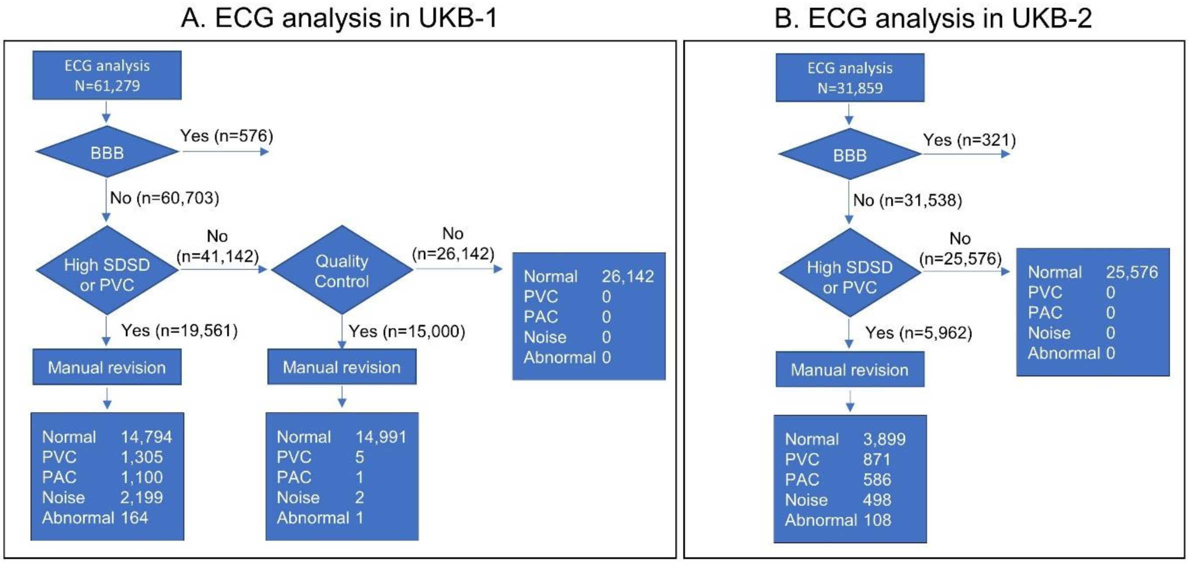

Supplementary Figure 1: Description of ECG data analysis for UKB-1 and UKB-2. BBB: Bundle-branch block morphology. SDSD: Standard deviation of successive RR-interval differences, used to measure heart rate irregularity. Normal: ECG shows normal morphology and sinus rhythm; PVC: Premature ventricular contraction; PAC: Premature atrial contraction; Noise: ECG signal quality too low to distinguish between sinus rhythm and PAV/PVC; Abnormal: Sinus node disfunction or atrial fibrillation.

Supplementary Figure S2

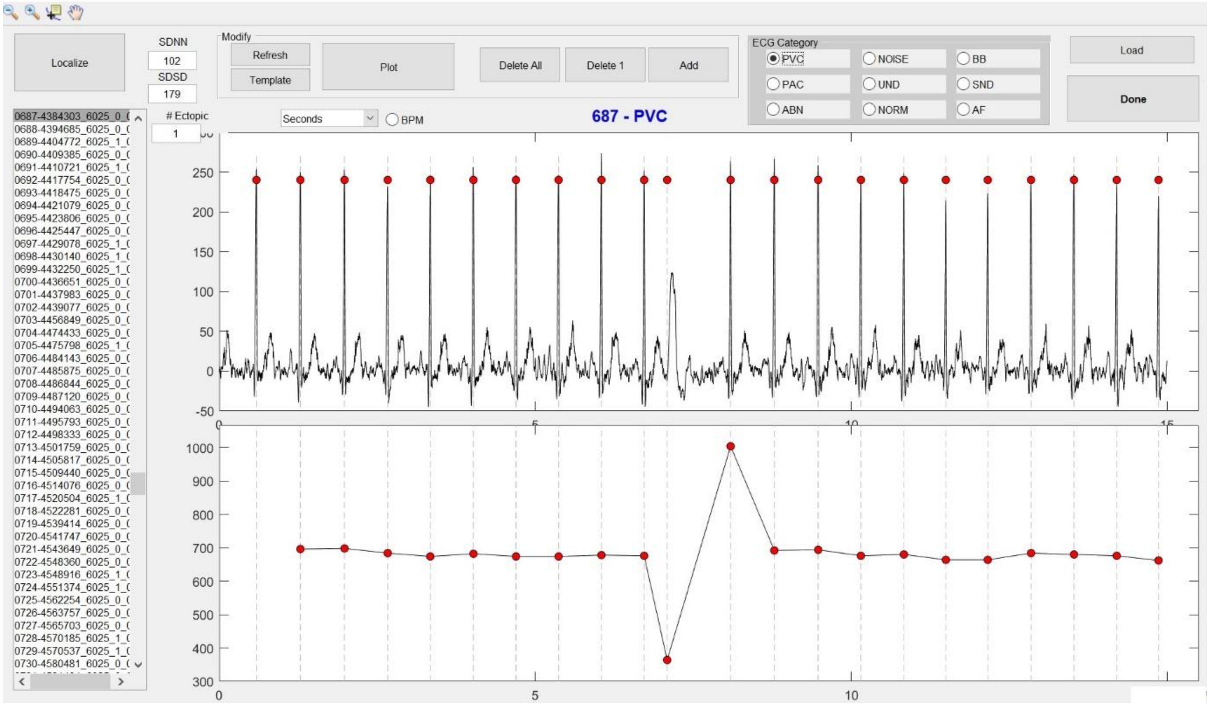

Supplementary Figure 2: The Graphical User Interface for signal annotation.

Supplementary Methods

Automatic detection of premature ventricular contractions

We implemented a sequence-to-sequence neural network using Convolutional Neural Network (CNN) layers for feature extraction on input data combined with recurrent neural network (LSTM) to support sequence prediction. The algorithm was implemented using the Keras framework with a Tensorflow backend and Python. For computational efficiency, we down sampled ECGs from 500 to 250 Hz. We then computed the short-time Fourier transform and used the data in the 0.05-45.00 Hz frequency range as input for the network to predict for each time-sample in the ECG whether it belonged to (1) A normal (narrow) QRS complex; (2) A QRS complex with premature ventricular contraction (PVC) morphology; (2) Neither of them (i.e. no QRS complex).

The network was composed of 2 blocks, the first block consisted of two 1D convolutional layers, each of them followed by a Relu activation layer. The output of this block was connected to a dropout layer to avoid over-fitting. The output was fed to the LSTM layer and then a dense fully connected layer to interpret the features extracted by the LSTM. Finally, the output was fed into a dense layer with three classes (normal QRS complex, PVC QRS complex, and no QRS complex), which was activated using a 'Softmax' function. The network was trained in 723 curated ECGs that were selected based on RR interval patterns suggesting the presence of ectopic beats. Data was split in a training and testing set with an 80:20 ratio to optimise hyperparameters such as batch and step size. We evaluated the performance using k=10-fold validation. The network showed the following performance to detect PVCs: F1= 91.8(+/- 1.8)%, precision = 91.8 (+/- 2.3)%, and recall = 91.9 (+/- 2.6)%.
